# Relationship between sleep and sexual functioning in Indian females

**DOI:** 10.1192/j.eurpsy.2024.1599

**Published:** 2024-08-27

**Authors:** R. Tripathi, P. Deedwania, A. Koparkar

**Affiliations:** ^1^Psychiatry, All India Institute of Medical Sciences, Gorakhpur; ^2^gynaecology, All India Institute of Medical Sciences, New Delhi; ^3^Community and Family Medicine, All India Institute of Medical Sciences, Gorakhpur, India

## Abstract

**Introduction:**

Sexual dysfunction is a taboo. It is a subject in many countries that negatively affects quality of life and may often be responsible for psychopathological disturbances. There is a little research on effect of sleep on female sexual response and behaviour.

**Objectives:**

The aim of the study was to assess prevalence of sexual dysfunction and sleep problems in adult females visiting OBGY OPD in a tertiary health care institution in a developing country and to observe the correlation between both.

**Methods:**

A cross-sectional observational study was conducted in a tertiary health care center in India. Female Patients presenting to Department of Gynecology and Obstetrics for any complaints were assessed for their sexual functioning and sleep profile

**Results:**

The mean age of the sample was 33.5 (6.2) years. All the female participants were married. Most of the participants were housewife and were living in a joint family. Almost 27 percent of the sample reported having sleep problems. The median sleep latency was 30 (15,60) minutes. The subjective total sleep time was 356.5 (60.3) minutes. The mean PSQI score was 4(2,6). The mean FSFI score was 26 (3.2). More than 50 percent of the females reported mild sexual problems and 10 percent reported mild to moderate problems. Most of the females didn’t have any symptoms suggestive of depression. Only two females reported moderate depression.

**Conclusions:**

Depression and anxiety have more effect on sexual functioning.

**Disclosure of Interest:**

None Declared

